# A Review of Biomimetic Topographies and Their Role in Promoting Bone Formation and Osseointegration: Implications for Clinical Use

**DOI:** 10.3390/biomimetics7020046

**Published:** 2022-04-16

**Authors:** Michael B. Berger, Paul Slosar, Zvi Schwartz, David J. Cohen, Stuart B. Goodman, Paul A. Anderson, Barbara D. Boyan

**Affiliations:** 1 Department of Biomedical Engineering, Virginia Commonwealth University, Richmond, VA 23284, USA; bergermb@vcu.edu (M.B.B.); zschwartz@vcu.edu (Z.S.); djcohen@vcu.edu (D.J.C.); 2 SpineCare Medical Group, San Francisco, CA 94109, USA; pslosar@spinecare.com; 3 Department of Periodontics, University of Texas Health Science Center at San Antonio, San Antonio, TX 78229, USA; 4 Department of Orthopaedic Surgery, Stanford University, Stanford, CA 94063, USA; goodbone@stanford.edu; 5 Department of Orthopedics and Rehabilitation, University of Wisconsin School of Medicine and Public Health, Madison, WI 53705, USA; anderson@ortho.wisc.edu; 6 Wallace H. Coulter Department of Biomedical Engineering at Georgia Tech and Emory University, Georgia Institute of Technology, Atlanta, GA 30332, USA

**Keywords:** topography, bone, titanium, stem cells, osteoblasts, biomimicry

## Abstract

The use of metallic and polymeric materials for implants has been increasing over the past decade. This trend can be attributed to a variety of factors including a significant increase in basic science research focused on implant material characteristics and how various surface modifications may stimulate osseointegration and, ultimately, fusion. There are many interbody fusion devices and dental implants commercially available; however, detailed information about their surface properties, and the effects that various materials and surface modifications may have on osteogenesis, is lacking in the literature. While the concept of bone-implant osseointegration is a relatively recent addition to the spine fusion literature, there is a comparatively large body of literature related to dental implants. The purpose of this article is to summarize the science of surface modified bone-facing implants, focusing on biomimetic material chemistry and topography of titanium implants, to promote a better understanding of how these characteristics may impact bone formation and osseointegration. This manuscript has the following aspects: highlights the role of titanium and its alloys as potent osteoconductive bioactive materials; explores the importance of biomimetic surface topography at the macro-, micro- and nano-scale; summarizes how material surface design can influence osteogenesis and immune responses in vitro; focuses on the kinds of surface modifications that play a role in the process. Biomimetic surface modifications can be varied across many clinically available biomaterials, and the literature supports the hypothesis that those biomaterial surfaces that exhibit physical properties of bone resorption pits, such as roughness and complex hierarchical structures at the submicron and nanoscale, are more effective in supporting osteoblast differentiation in vitro and osteogenesis in vivo.

## 1. Introduction

Osseointegration is the robust biological cascade that occurs after a biomaterial is placed in or adjacent to native bone tissue. The process consists of the following: (1) hematoma formation following surgical trauma; (2) an innate immune response characterized by macrophages and neutrophils; (3) migration of bone marrow stromal cells (MSCs) and osteoprogenitor cells from the native bone toward the material surface and their subsequent differentiation into bone-forming osteoblasts; (4) fusion of monocytes to form multi-nucleated osteoclasts and remodeling of the newly synthesized primary bone; (5) formation of competent, structurally anchored mature bone [1].

The term “osseointegration” implies that a material becomes integrated with adjacent bone. It is a broad term and encompasses materials as disparate as autografts and allografts, demineralized bone matrix (DBM), synthetic bone graft substitutes, metals, ceramics and polymers. Osseointegration can occur in a physical sense when bone grows in from the native bone bed, stabilizing the implant. If the implant has a porous surface, the bone ingrowth may form a mechanical interlock [2,3].

Bone can also form directly on materials that possess specific chemical and/or physical properties. For autografts and allografts, these are achieved by the action of osteoclasts on the surface, leaving osteoclast resorption pits with micro-, meso-, and nanoscale textures as well as biochemical cues for MSC and osteoprogenitor cell recruitment, attachment and differentiation. Materials like DBM also provide factors that promote osteoblast differentiation, such as bone morphogenetic protein 2 (BMP2) [1,2]. The physical properties associated with osteoclast resorption pits can be generated on biomaterials using a variety of techniques, enhancing osseointegration through adsorption of various proteins, recruitment and attachment of MSCs and osteoprogenitor cells, and osteoblast differentiation, subsequent bone formation and downstream remodeling. While these events can be achieved using materials such as titanium and its alloys, not all materials support osteogenesis and osseointegration in this manner. For example, materials such as poly-ether-ether-ketone (PEEK) support formation of a fibrous connective tissue interface instead [4].

Successful osseointegration was first achieved in dental implant applications. The pursuit to provide a stable bone anchor for reconstructing a functional dentition is facilitated by a variety of surface modifications, leading to bone growth onto the surface. Bone-facing implants are also used in orthopedics. For example, stability of joint prostheses is achieved through a mechanical interlock resulting from bony ingrowth into a microporous surface topography. Alternatively, orthopedic surgeons may use polymethylmethacrylate to cement the prosthesis within the medullary canal, which negates the value of any surface modification for enhancing bone ingrowth.

Implants are used in spinal fusion applications in order to eliminate pathologic motion between two or more vertebral bodies. This is commonly achieved by removing the intervertebral disc and replacing it with an interbody fusion device (IBFD). IBFDs are developed uniquely for each anatomical location within the spine and, depending on function and loading requirements, may provide a geometry for which bone graft materials can be placed within or adjacent to the implant [5]. In addition, the IBFD provides structural support to withstand the compressive forces of the disc space to maintain vertebral alignment [6]. Advancements in surface processing, similar to those used for dental implants, have developed biomimetic implant surfaces with irregular structures to further facilitate fusion by enhancing the differentiation of MSCs and stimulating the production of osteogenic soluble signaling factors [7]. Together, graft containment, structural support, and biomimetic surface modification facilitate the eventual fusion of bone across the disc space.

The concept of implant osseointegration with the vertebral body is a relatively recent addition to the spine fusion literature, and implants are rapidly being developed and modified in a variety of ways to try to promote bone formation. Some of the approaches used to modify the surfaces of IBFDs come directly from the literature on endosseous dental implant applications, which have focused on evolving materials and surface modifications in order to optimize rapid and reliable integration of an implant with host bone. Therefore, the overall goal of this article is to provide a perspective on IBFD surface design, based on the available dental implant material science literature and clinical oral implant research with respect to existing biomimetic surface processing.

## 2. Spine Fusion Devices as a Subset of Bone-Facing Implants

Historically, autografts were used as bone fillers in periodontology and as spacers between vertebral bodies to stimulate ossification and fusion in anterior cervical discectomies and fusion (ACDFs). However, due to major morbidity from the donor sites, clinicians have transitioned to allografts and synthetic materials as these technologies have matured. While some surgeons still use allograft bone as their implant of choice, a majority of fusions after ACDF are performed by inserting implants made of polymer materials or titanium alloy. Titanium was the first synthetic material used in vertebral fusion dating back to the late 1970s. These implants were first iterations and were chosen due to their high wear resistance, excellent biocompatibility, and osteoconductivity demonstrated from use as dental implants. One limitation of these Ti-based implants was the mismatch in mechanical modulus between titanium and the native bone tissue leading to stress shielding and increasing the potential for subsidence, or vertebral spacing loss. These implants lacked discrete, specific surface properties engineered to direct cellular response, possessing instead a less sophisticated topography [5,8].

In the 1990s, polymers became attractive due to their more similar mechanical modulus and radiolucency, making it possible to observe fused bone on plane X-rays. PEEK is the primary polymeric-material used in these devices and PEEK IBFDs have dominated the market for the last 25 years. Chemically, these IBFDs were considered biologically “inert” in short-term analyses because bone did not form in close approximation to the implant, and the nature of the smooth surface and hydrophobicity resulted in limited osteoconductivity [9]. Recognition that PEEK implants are surrounded by a fibrous connective tissue has shown that these materials are not “inert.” Rather, they support fibrosis at the interface with bone. To overcome this, PEEK IBFDs are increasingly being coated with titanium, to attempt to provide the same biocompatible properties as titanium-based IBFDs, and clinical evidence has shown some promise, however, many of these IBFDs lack biomimetic surface topographies [10,11].

Clinical assessment of spinal fusion rate and subsidence has been assessed in multiple meta-analyses and retrospective studies. Earlier studies compared smooth PEEK and Ti-based cages and found increased incidences of subsidence in the Ti IBFDs. Other studies have shown that cage footprint area, age, bone density, and excessive grafting material, not IBFD material, is a cause for these differences [12,13]. Recent meta-analyses have shown that PEEK cages exhibit either no difference in fusion rates or statistically significantly less fusion compared to Ti-based counterparts, and no differences in subsidence and improved clinical outcomes after 1 year, as clinical approaches have been refined and cage design has been improved [5,13,14,15].

There is growing scientific research into the long-term effects of both metals and polymers in the human body. Metal allergy and immune sensitization is a concern for many orthopedic and dental implants, specifically around the generation of small micron-scale particles in the peri-implant environment inducing an immune response, usually a result of corrosion or wear [16,17]. Interestingly, there is evidence specifically with PEEK polymer indicating that the bulk polymeric materials can elicit inflammatory immune responses from the innate immune system, which helps to promote the formation of a fibrous connective tissue interface between the host bone and IBFD, leading to implant instability [18,19,20]. Several cases of non-union in polymer-based implants exhibited severe osteolysis, posing a risk to patients during revision [5]. This may be a factor in the growing use of IBFDs manufactured from, or coated with, titanium alloys, particularly titanium-aluminum-vanadium alloy (Ti6Al4V). In 2010, Ti-based implants comprised roughly 5% of the global market, but this has grown to 46% of the market in 2019, while PEEK’s market share has declined from 68% to roughly 44% in 2019 [21]. The trend to use Ti6Al4V can be attributed to a variety of factors including a significant increase in basic science research focused on the biomimetic material characteristics of implants and how various surface modifications may stimulate implant osseointegration and fusion [4,22].

## 3. Methodology of Literature Search

Articles indexed online at National Library of Medicine (PubMed.gov) and online using Google Scholar were inquired for basic research and clinical articles during or after 1995. Keywords used in these searches were one or a combination of the following: titanium, PEEK, interbody fusion device, IBFD, spine cage, osseointegration, osteoblast, microroughness, topography, nanoscale, nano roughness, osteoclast, surface modification, surface properties, grit blast, acid etch, titanium plasma spray, 3D printing, additive manufacturing, immune, and bone. Journals whose scope of research focuses on evaluating bioactive materials, osseointegration, dental implantology, interbody fusion, and tissue engineering were selected for review and compilation. Historical perspective was provided by landmark studies from Per-Ingvar Branemark prior to 1995.

## 4. Biomimetic Nature of Titanium and Its Alloys

Branemark first reported on the biocompatibility of commercially pure Ti, noting that it was an ideal material for use in bone [23,24]. The reason for this was the natural passivation layer composed of titanium dioxide (TiO_2_) ceramic, which forms on Ti and its alloys when they are exposed to air. The TiO_2_ ceramic surface is well tolerated in bone and does not cause a fibrous connective tissue interface to form. Ti was quickly adopted by the dental implant industry because of its mechanical similarity to bone, compared to other metals, and is becoming the choice material for craniofacial reconstruction [25]. Roughened surfaces were shown to be more effective than a smooth machined or polished surface, and there was soon a number of dental implant designs that had irregularly oriented, microtextured surfaces that were generated by a variety of methods.

One of these methods, Ti plasma spray (TPS), produced roughness that projected out from the implant surface. TPS was highly irregular in form, but it stimulated bone-forming osteoblasts to become well differentiated in vitro [26] and supported osteogenesis in vivo [26]. TPS also had drawbacks. The knobs on the ends of the branched roughness fractured off, leading to leakage of Ti ions underlying the TiO_2_ layer, which were taken up by the surrounding cells [27,28]. In vitro studies showed that osteoprogenitor cells attached to the TPS surface, but their attachment and proliferation were reduced compared to other surface topographies, so the surface of the Ti substrates was not well covered [29]. In the end, the TPS implants were replaced in the marketplace by implants that had a more stable and improved surface topography.

The newer approaches involved grit-blasting the surface followed by an acid treatment to remove any residual grit. Depending on the chemistry of the grit (aluminum oxide, calcium phosphate, magnesium sulfate, etc.), the size of the grit, the power and the length of the sandblasting process, the Ti surface is left with pits and craters of various sizes [27]. This “roughness” was in the scale of hundreds of microns to tens of microns. The use of acids provided another broad range of micro- and meso-scale surface topographies, varying with the type of acid used, the temperature of the acid, and the length of exposure. This grit-blasting/acid-etching approach resulted in Ti implant surfaces that had macroscale, microscale and mesoscale structures [4].

**Figure 1:**
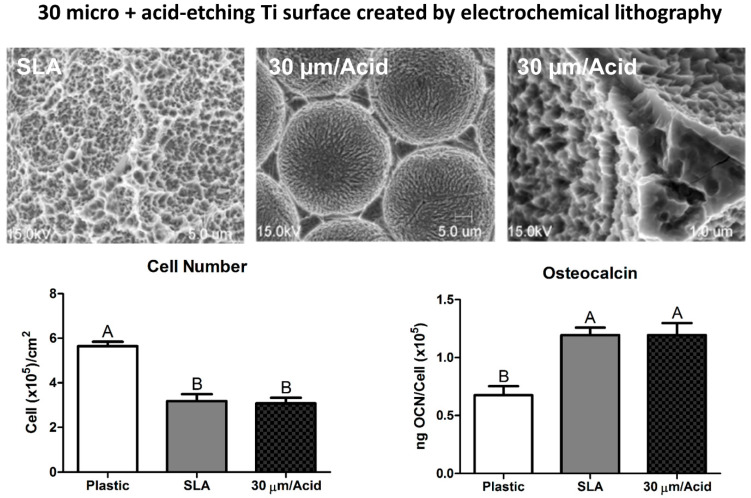
Osteoprogenitor cells differentiate in response to discrete surface topography that mimics the physical parameters of a bone surface modified by a bone resorbing osteoclast [30]. Clinically relevant implant surfaces were produced by grit blasting with large grit corundum and subsequently acid etching in the same manner. Osteoprogenitor cells cultured on both these modified titanium substrates exhibited increased osteoblastic differentiation, as seen by decreased proliferation and increases in osteoblastic markers such as osteocalcin, compared to cells cultured on tissue culture plastic. Groups not sharing letters are significant at a *p*-value of <0.05.

Most of these methods resulted in a surface that had a complex multi-scale roughness, characterized by craters, peaks, and valleys. To obtain a better understanding of how cells recognized these different surface topographies, test surfaces were made by electron photolithography, using the same method that is used to manufacture microchips. These substrates were coated with a nano-thin layer of Ti and had craters varying in size from 5 to 100 μm, which were placed either next to each other or separated by a defined space. They were then either acid etched or anodized producing an average roughness about 700 nm in height [30]. Osteoprogenitor cells cultured on surfaces that had adjacent 30 micrometer craters with an acid etched meso-scale and nano-scale topography exhibited statistically superior differentiated phenotypes to surfaces that did not possess these features (Figure 1) [30,31]. This same enhancement of osteoblast differentiation was observed on Ti substrates that were sandblasted and acid etched, creating a complex macro/micro/meso-scale roughness.

**Figure 2:**
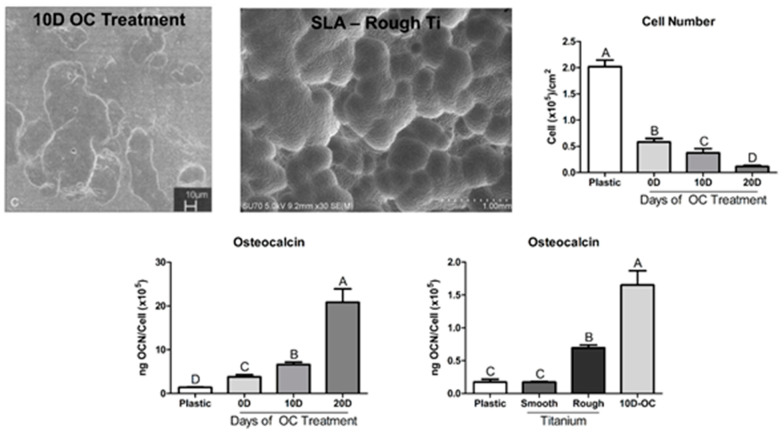
Osteoclast treatment of bone wafers conditions the surface of the bone to stimulate osteoblastic differentiation of osteoprogenitor cells [34]. Scanning electron imaging of an osteoclast modified bone surface at 10 days shows topographical alterations as the mineral is resorbed away. Culturing cells on bone wafers increases osteoblastic differentiation without modification by osteoclasts and increasing the length of surface modification by bone resorbing osteoclasts increased the differentiation of osteoprogenitor cells and osteocalcin production. Groups not sharing letters are significant at a *p*-value of <0.05.

Scanning electron microscopy of osteoclast resorption pits indicate that these specific structural characteristics are also found on bone surfaces in vivo after they have been conditioned by osteoclasts [32,33]. In vitro experiments examining the response of osteoprogenitor cells to osteoclast-resorbed bone wafers show that the osteogenic stimulus of the surface increases with the degree of resorption [34]. In comparison to their behavior on tissue culture polystyrene, osteoprogenitor cells cultured on the biomimetic sandblasted/acid etched Ti substrates exhibit significantly greater osteoblast differentiation. However, this effect is not as robust as the response of these cells to an osteoclast-resorbed bone surface (Figure 2).

When osteoclasts condition bone, they not only resorb mineral, but they also modify the chemistry of the bone resorption pit in order to recruit osteoprogenitor cells and induce new bone formation, and synthetic materials that contain this chemical modifications have shown ability to regulate cell response [35]. While the chemical information that they leave behind is important, the physical alterations that they leave behind are critical features when designing a biomimetic implant surface. The ability to now replicate and commercially manufacture material surfaces that are naturally occurring (biomimicry) has opened to door to the concept that Ti alloys can be used effectively in orthopedics and that the kinds of surface modifications that have been so successful in dental implantology can be applied to skeletal bone.

## 5. Biomimicry: Nanotopography as a Critical Variable in Surface Topography

All surfaces exhibit a nanotopography to some extent. The concept of nanotopography has emerged in the biomaterials literature to refer to structural features that have at least one dimension that is less than 100 nm in diameter [36]. Nanotextures can be generated by a variety of methods, including acid etching, oxidation, and addition of nanoparticles and nanotubes [36,37,38,39]. In most instances, the resulting features are actually mesoscale, meaning that they are less than 1 μm but greater than 100 nm, although they may have a dimension that is less than 100 nm.

A number of in vitro studies have been performed to assess if these surface topography manipulations can promote cellular reactions stimulatory for bone formation. In some cases, surface modifications can enhance the osteogenic properties of Ti and Ti-alloy substrates [40], but careful comparative analysis shows that this is technique sensitive and is limited to only a subset of modifications [41]. The variation in response is due to the resulting shape and chemistry of the nanotexture and its relationship to other physical features of the surface [42]. The most effective biomimetic surfaces for osseointegration have been shown to have craters that are between 30 and 100 μm in diameter, overlaid with pits that are 1–3 μm in diameter and a nanotexture that has a mesoscale dimension of 500–700 nanometers with an “isosceles triangle” morphology [7].

A variety of claims have been made concerning nanoscale surface modifications on Ti implants. In some models (additive processing) the particles are added or attached in some way to a machined surface or a 3D printed surface [36,43]. These technologies are often tested in conventional cell culture dishes and use osteogenic media to stimulate osteoblast differentiation of MSCs as a test for their ability to stimulate osteogenesis in vivo. These tests rely on the properties of the media supplements (high Ca++, dexamethasone, and beta-glycerol phosphate, as well as bone morphogenetic protein 2 [BMP2]) to ensure that mineral deposition can occur, but the outcomes may reflect the supplements rather than the actual topographic elements [44,45,46].

In contrast, when MSCs are cultured on osteoclast resorption pit biomimetic substrates that have specific micro-/meso-/nano-textured surfaces produced via subtractive processing (grit blasting/acid etching), osteoblast differentiation occurs rapidly even in the absence of these media supplements, indicating that it is the complex topography of surface that is stimulating the osteogenic outcome [7,47]. This is supported by in vivo data, both in animal models and in humans. In humans, a 10-year study evaluating 511 dental implants with micron and submicron scale roughness demonstrated 98.9% implant survival rate and healthy soft-tissue in patients who previously presented with peri-implant inflammation [48]. In animals, micro-/nano-roughened implants were able to overcome the effects of post-menopausal osteoporosis using aged, ovariectomized rats without additional pharmacologic intervention, and increased wettability at the time of placement further increased bone formation peri-implant [49,50]. This effect of wettability is also seen in humans, where bone formation was improved 2 and 4 weeks post implantation (14.8% vs. 12.2% and 48.3% vs. 32.4%, respectively) compared to microroughened implants alone but were equal by 6 weeks (61.5%) [51]. These data demonstrate the best physiological responses occur on surfaces possessing a combination of all of these topographic properties, specifically structures at the macro-/micro-/meso-/nano-scale.

As noted above, the number of possible surface modifications is large, but not all resulting surface topographies are clinically useful, or even desirable. Determining whether a surface feature will have a positive impact on clinical outcomes, particularly in patients with underlying conditions affecting bone health, requires comprehensive in vitro and in vivo assessments. It is critical that the surface characteristics be extensively analyzed, as treatment steps during processing can modify all length scales. This is especially important as the industry moves to additively manufactured implant designs [43]. While it is attractive to produce complex three-dimensional structures that address vertebral anatomy, the methods used to fabricate the IBFD and the post-build processing can all impact the eventual surface topography. Until these surfaces are evaluated in vitro and preclinically it is difficult to fully predict the response of cells or systems to these synthetic biomaterials.

## 6. Biomimetic Surface Topography and Immune Modulation

There is increasing interest in immunomodulation and its role in biological responses to biomaterials. Early studies showed that maturation of dendritic cells is sensitive to Ti implant surface properties [52], suggesting that immune cells present in the environment following implant placement could be influenced and, in turn, influence the clinical outcome. Recently, studies have also demonstrated that recruitment of circulating neutrophils and the capturing of these cells from the vasculature is regulated by implant surface properties and may be a key determinant in cell recruitment and resolution of inflammatory signaling [53,54].

Subsequent studies have supported this hypothesis by showing the Ti implants with osteoclast resorption pit biomimetic surfaces result in the differentiation of macrophages along a pro-healing M2 pathway whereas surfaces that have a comparatively smooth surface topography result in a pro-inflammatory M1 phenotype [55,56]. Histopathology of tissues confirm that this is the case in vivo as well. Adaptive immune cells are also regulated by implant properties, and biomimetic topographies have been shown to maintain the immaturity of dendritic cells and alter T cell and helper cell activation and regulation in response to topography and wettability [55,57].

Factors produced by MSCs in response to surface topography indicate that there is cross talk between immunomodulators generated in response to surface topography with those factors produced by immune cells. In general, MSCs exhibit increased production of interleukin 10, interleukin 4 and reduced production of factors associated with apoptosis, necrosis, and chronic inflammation such as interleukin 1 and interleukin 6 when they are cultured on biomimetic Ti, TiZr, and Ti-6Al-4V substrates [42,58,59].

Implants that comprise alternative alloys or polymeric materials, such as PEEK, have been further modified to have surface properties similar to that of Ti and its alloys, either through the addition of layers of Ti or by the incorporation of these alloys, or other additives into the bulk substrate [60,61]. In some instances, surface modification improves the overall cellular response of these implants compared to machined or untreated surfaces. However, when compared to Ti, these alternative implant options create a more inflammatory environment, resulting in the production of inflammatory cytokines and a mixed pool of macrophages with both M1 and M2 polarizations, and were correlated with lesser bone formation at 10 days, suggesting the switch to regenerative macrophages is delayed [9]. Overall, the use of additives and creation of surface composites to overcome the natural inability of bone to form on a polymer surface possesses the potential risk of a fibrous tissue interface, as shown in preclinical models and in the clinic.

## 7. Conclusions

This overview of how material surface design can influence osteogenesis and immune responses in vitro has focused on the kinds of surface modifications that play a role in the process. These modifications can be achieved through a variety of methods on two-dimensional surfaces, but the literature supports the hypothesis that those surfaces that exhibit physical properties of osteoclast resorption pits are more effective in supporting osteoblast differentiation in vitro and osteogenesis in vivo. The increasing use of advanced manufacturing to produce implants with complex three-dimensional structures may necessitate the development of new ways of creating these biomimetic topographies [43,62].

## Data Availability

Not applicable.
